# Advancements and challenges in CAR-T cell therapy for solid tumors: A comprehensive review of antigen targets, strategies, and future directions

**DOI:** 10.1186/s12935-025-03938-0

**Published:** 2025-08-23

**Authors:** Jiajun Zhu, Jianming Zhou, Yiting Tang, Ruotong Huang, Chengjia Lu, Ke Qian, Qingyu Zhou, Jingjun Zhang, Xiaoyi Yang, Wenhan Zhou, Jiaqiang Wu, Qiudan Chen, Yong Lin, Shuying Chen

**Affiliations:** 1 Department of Laboratory Medicine, Huashan Hospital, Fudan University, 12 Wulumuqi Middle Road, Shanghai, 200040 China; 2 Shanghai Medical College, Fudan University, 130 Dongan Road, Shanghai, 200032 China; 3 Department of Clinical Laboratory, Central Laboratory, Jing’an District Center Hospital of Shanghai, Fudan University, 259 Xikang Road, Shanghai, 200040 China; 4 Department of Rehabilitation Medicine, The Sixth People’s Hospital, Shanghai Jiao Tong University School of Medicine, Shanghai, 200233 China; 5 Department of Dermatology, Huashan Hospital, Fudan University, 12 Wulumuqi Middle Road, Shanghai, 200040 China; 6 Department of Clinical Laboratory, Fifth People’s Hospital, Fudan University, 801 Heqing Road, Shanghai, 200240 China

**Keywords:** CAR-T, Solid tumor, Immunotherapy, Antigen target, Personalized treatment

## Abstract

Chimeric antigen receptor-modified T cell therapy, originally employed in hematological malignancies treatment, has made significant strides in addressing solid tumors in recent years. Presently, second-generation CAR-T therapy has reached clinical implementation, while fifth-generation CAR-T therapy is in active development. However, initial clinical trials in solid tumors have shown limited success, primarily due to the immunosuppressive microenvironment of the tumor and the scarcity of tumor-specific antigens. Nevertheless, emerging strategies, including combination therapies and gene editing technologies, exhibit promising potential in enhancing CAR-T cell effectiveness. In this review, we comprehensively examine the current state of research on CAR-T cell therapy for solid tumors, with a particular focus on the various antigenic targets that have been explored for solid tumors. We critically review established and novel targets, providing insights into their therapeutic potential and limitations. Additionally, we highlight the key challenges that currently hinder the success of CAR-T therapy in solid tumors, including the choice of target antigens, physical barriers, antigen escape, immunosuppressive microenvironment, and adverse reactions. Despite the formidable challenges confronting CAR-T cell therapy in solid tumors, ongoing research is forging a path towards its integration into the mainstream cancer treatment paradigm, offering renewed optimism for enhanced outcomes in patients afflicted with solid malignancies.

## Introduction

Chimeric antigen receptor-modified T cell (CAR-T) therapy, a form of precision-targeted treatment for tumors, stands out as a forefront area in contemporary clinical oncology. It has emerged as one of the most extensively studied fields in recent years for the treatment of tumors. It is undoubtedly a very efficient, accurate and promising immunotherapeutic approach to treat tumors. Though it was originally employed in leukemia and lymphoma treatmen [1, 2], it is now confirmed to be promising in treating solid tumors.

### Development of CAR-T therapy

**Fig. 1:**
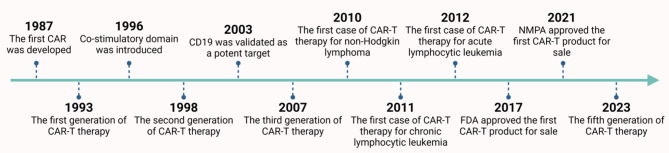
Key events in the history of CAR-T therapy

Since 1987, Kuwana et al. first proposed the first generation of chimeric antigen receptor (CAR) by designing chimeric receptor composed of immunoglobulin-derived V regions and T-cell receptor-derived C regions [3]. Over the next 30 years, with continuous development and improvement, several generations of CAR have been constructed. In 2017, CD19 CAR-T cells received FDA approval for the treatment of leukemia and lymphoma. In 2021, Axicabtagene Ciloleucel Injection and Relmacabtagene autoleucel injection were successively approved for listing in China. The history of CAR-T is summarized in Fig. 1. So far, CAR-T cell therapy have played a significant role in B-cell malignancies, revolutionizing the field of tumor immunotherapy. However, Early attempts of second-generation CAR-T technology in solid tumors have met with setbacks, and most of the related drugs for CAR-T treatment in solid tumors are still under development [1]. 

### The five generations of CAR-T therapy

**Fig. 2:**
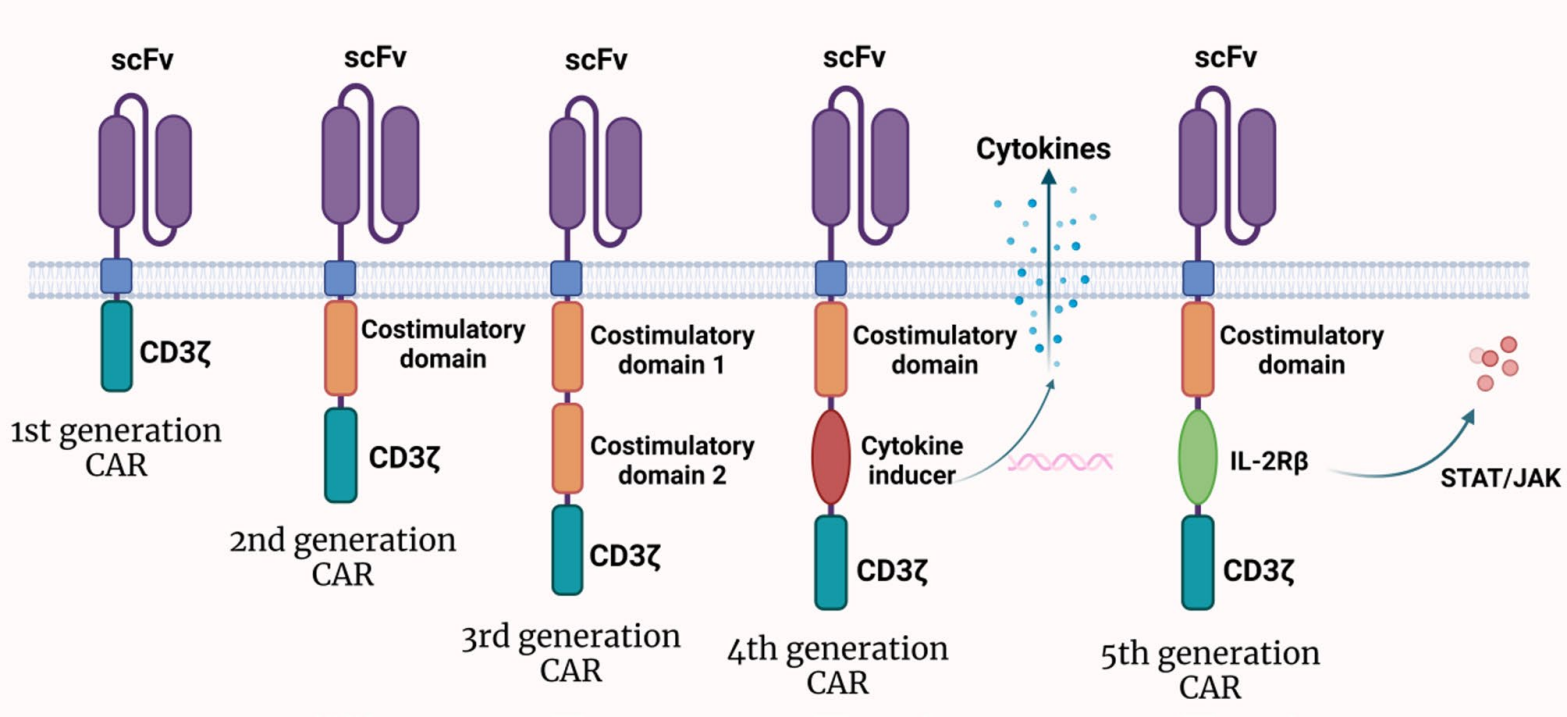
Five generations of CAR. This figure provides a detailed comparison of the five generations of CAR-T therapy. It shows the progression from the first-generation CAR with a single CD3ζ signaling domain to the second and third generations incorporating co-stimulatory signals (e.g., CD28, 4-1BB) for enhanced T cell activation and proliferation. The fourth generation (TRUCKs) introduces cytokine secretion to further boost antitumor effects, while the fifth generation enhances intracellular signaling through cytokine receptors (e.g., IL-2Rβ) to improve cell function. The figure also depicts how each generation performs upon encountering target antigens, highlighting differences in activation, proliferation, and cytotoxic capabilities

Up to now, CAR-T therapy has experienced five generations of drug development and has made remarkable progress in Fig. 2. The first generation of CAR-T technology contained only one intracellular signaling domain (CD3ζ) [4]. The development of the second and third generations of CAR-T involved the incorporation of one or two co-stimulatory signal domains (such as CD28, OX40, and 4-1BB) to the first-generation CAR, respectively [5–7]. The fourth generation of CAR (TRUCKs)is to further introduce functional cytokines to enhance the killing and expansion ability of T cells [8]. The fifth generation of CAR enhances the intracellular domain of the cytokine receptor (IL-2Rβ) on the basis of the second generation, further enhancing cell proliferation and activation [9, 10]. It can be seen that since the advent of the fourth generation of CAR-T, researches have gradually focused on giving this therapy a wider range of anti-tumor capabilities, so that it can be applied to the treatment of various solid tumors. With the development of each generation of CAR-T therapy, the potential of CAR-T in the treatment of solid tumors is becoming more evident.

### Common antigen targets in solid tumors

**Fig. 3:**
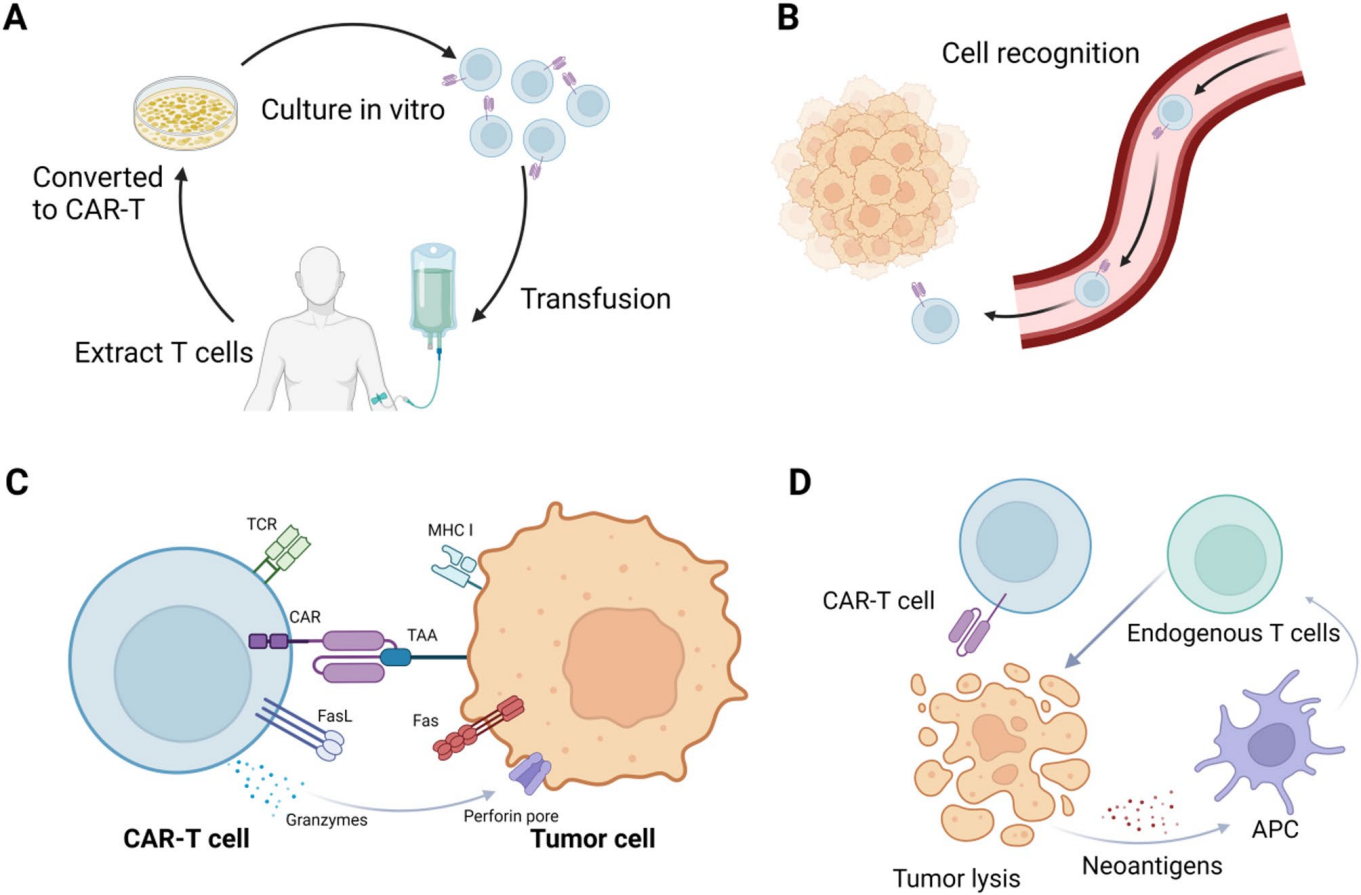
This figure illustrates the comprehensive mechanism of CAR-T cell therapy. Panel (**A**) shows the in vitro culture process, where T cells are extracted from the patient, genetically modified to express chimeric antigen receptors (CARs), and expanded in the laboratory to achieve a sufficient number for infusion. Panel (**B**) depicts how these engineered CAR-T cells travel through the bloodstream and specifically recognize tumor antigens on cancer cells, enabling them to localize to the tumor site. Panel (**C**) details the interaction between CAR-T cells and tumor cells, where the binding of the CAR to the target antigen activates the T cell, leading to the release of cytotoxic granules that induce apoptosis in the tumor cell. Finally, panel (**D**) highlights the broader immune response, where activated CAR-T cells recruit additional immune cells, participate in antigen presentation, and enhance the overall antitumor effect through collaborative immune activity

The anti-solid tumor mechanism of CAR-T cells is similar to that of non-solid tumor (Fig. 3), but they face completely different situations. Over the past few years, notable progress has been made in CAR-T therapy treating solid tumors. Following the achievement of a successful binding to the CD19 target in B cell acute lymphoblastic leukemia and diffuse large B-cell lymphoma, researchers have also identified more effective binding targets in solid tumors. With the development of sequencing techniques, a variety of characteristic solid tumor targets are being discovered. Common solid tumor targets include MSLN, GPC3, GD2, HER2, CEA, DLL3, BCMA, GUCY2C, EGFR, NKG2DL and so on. Corresponding CAR-T therapy drugs targeting some of the receptors listed in Table 1 have already entered preclinical and clinical trials. The following section will provide a detailed overview of the latest studies based on different targets.

**Table 1:** Common target antigens of solid tumors

Tissues	Targeted antigens	Investigational Stage
Neural	GD2 [11]	Phase II/III
	L1CAM [12]	Preclinical
	PHOX2B [13]	Preclinical
	CAIX [14]	Phase I/II
Brain	EGFR VIII [15]	Phase II/III
	HER2 [16]	Phase I/II
	IL13RA [16]	Phase I/II
Lung	CEA [17]	Phase I/II
	EGFR [18]	Phase III/IV
	HER2 [19]	Phase I/II
	MSLN [20]	Phase II/III
	EPCAM [21]	Preclinical
	CDH17 [22]	Preclinical
Liver	GPC3 [23, 24]	Phase I/II
	CEA [25]	Phase I/II
Colorectum	CEA [26]	Phase I/II
	CDH17 [27]	Preclinical
	GUCY2C [28]	Phase I/II
	CD44V6 [29]	Preclinical
	NKG2D [30]	Preclinical
Gastric	CEA [31]	Phase I/II
	HER2 [32]	Phase I/II
	CLDN18.2 [32]	Phase II/III
	MSLN [33]	Phase II/III
Renal	VEGFR2 [33]	Phase I/II
	CAIX [34, 35]	Phase I/II
Ovarian	FRα [34, 35]	Phase I/II
	CEA [31]	Phase I/II
	HER2 [36]	Phase I/II
	MSLN [37]	Phase II/III
	L1CAM [38]	Preclinical
	MUC16[39]	Phase I/II
Pancreas	CEA	Phase I/II
	MSLN [40, 41]	Phase II/III
	MUC1 [42]	Preclinical
	CLDN18.2 [43]	Phase II/III
Prostate	PSMA [44]	Phase I/II
	PSCA [45]	Preclinical
Breast	CEA [46]	Phase I/II
	cMET [47]	Preclinical
	HER2 [48]	Phase I/II
	MSLN [49]	Phase II/III
	MUC1 [50]	Preclinical
Skin	GD2 [51]	Phase II/III
	VEGFR [51]	Phase I/II
Soft tissue	GD2 [52]	Phase II/III
	HER2 [53]	Phase I/II
Head and neck	ERBB family [54]	Phase I/II

### Mesothelin (MSLN)

**Fig. 4:**
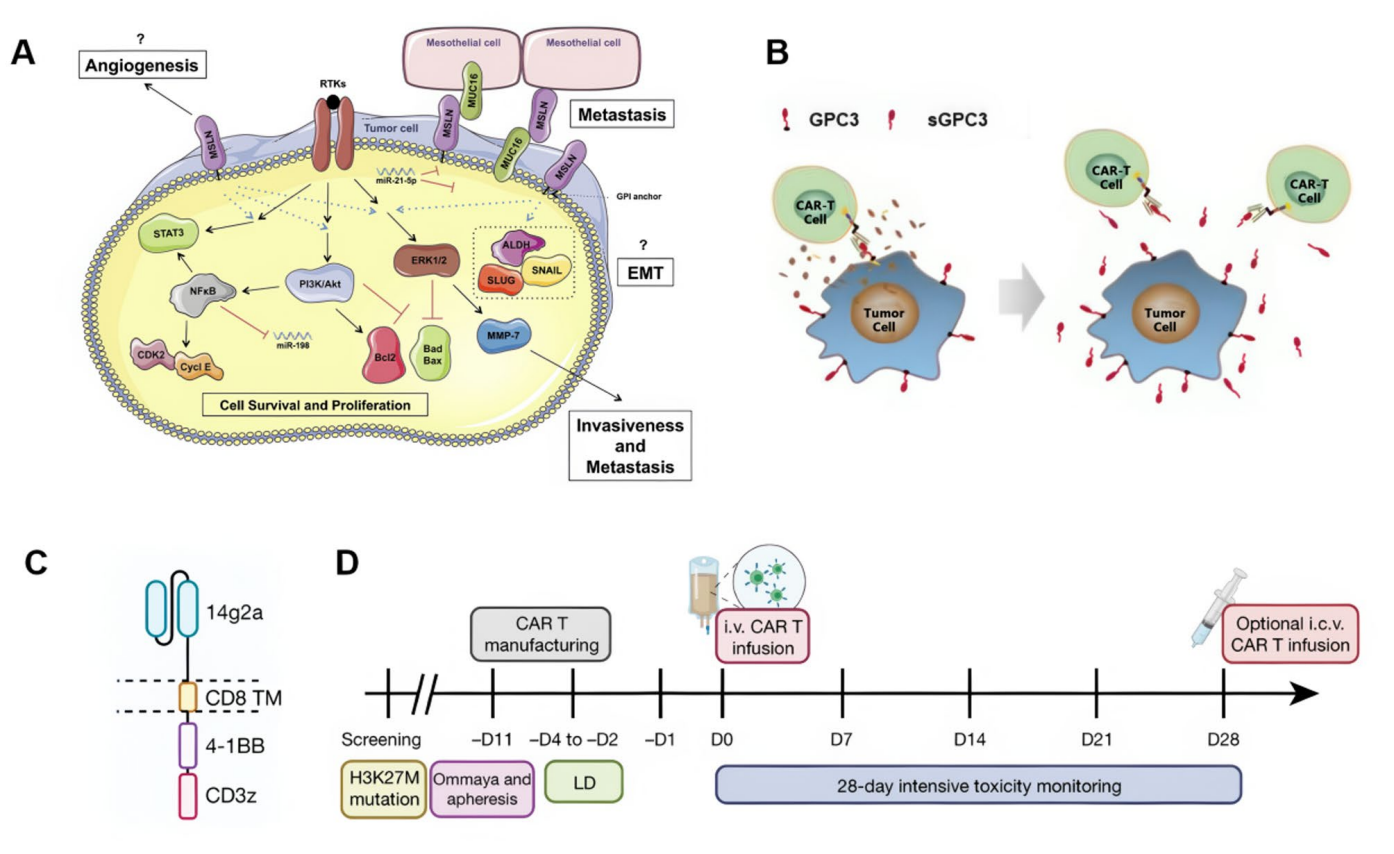
The therapeutic principle and process of CAR-T cells. **(A)** Putative roles of MSLN in PDAC progression [55]. Copyright 2020 by the authors. Licensee MDPI, Basel, Switzerland. **(B)** Proposed working model of sGPC3 in GPC3- specific CAR-T cell therapy [24]. Reproduced with permission. Copyright 2021, BMJ Publishing Group Ltd & Society for Immunotherapy of Cancer. **(C)** GD2-4-1BB-CD3ΖCAR schematic.TM, transmembrane domain [64]. **D** Outline of clinical trial design [64]. Copyright 2022 The Authors. Licensee CC BY 4.0. All these figures are not modified

MSLN functions as a glycoprotein on the cell surface, which is widely expressed in tumors such as malignant pleural mesothelioma [54], pancreatic cancer [55], ovarian cancer [56], some lung cancer and so on. Elevated MSLN expression intricately modulates multiple cellular signaling pathways and is strongly associated with tumor proliferation, invasion, and unfavorable prognosis (Fig. 4A) [57]. In 2019, Zhang et al. identified that region III of MSLN targeted CAR-T cells mediated strong antitumor responses in the gastric cancer NSG mice model and effectively reduced the in vivo growth of large ovarian tumors [58]. In 2023, Schoutrop et al. assessed two traditional second-generation MSLN-CAR T cell designs through preclinical in vitro and in vivo models of ovarian cancer. Their investigation demonstrated that precisely modulating the activation of MSLN-CAR T cells resulted in enhanced and superior antitumor responses within the context of ovarian cancer models [59]. Meanwhile, several methods to enhance therapeutic effect of MSLN targeted CAR-T cells have been discovered these years. A recent study proves that Irinotecan can significantly augment the antitumor efficacy of MSLN-targeted CAR T cells, offering a promising strategy for combination therapy in MSLN-positive solid tumors [60]. However, in 2022, Chen et al. conducted an investigator-initiated clinical study to evaluate the safety and efficacy of anti-MSLN CAR-T cell therapy in patients with ovarian cancer. This study evaluates anti-MSLN CAR-T therapy for ovarian cancer. Preclinical models reveal strong antitumor activity. A phase I trial on three patients shows tolerability and efficacy, with one achieving stable disease and another a partial response [37]. 

### Glypican-3(GPC3)

GPC3, a glycoprotein present on the surface of the cell, exhibits elevated pathological expression in hepatocellular carcinoma (HCC). Despite this, conventional GPC3-targeted CAR-T therapies have demonstrated efficacy in only a limited subset of HCC patients. In 2020, Sun et al. developed two variants of CAR-T cells, each targeting distinct epitopes of GPC3. Their study demonstrated that the presence of sGPC3 significantly impeded cytokine release and the cytotoxicity of anti-GPC3 CAR-T cells in vitro, unveiling a novel mechanism of immune evasion in HCC(Fig. 4B) [24]. Ma et al. confirmed the enhanced targeting capability of CAR-T cell membrane-coated nanoparticles in comparison to mesoporous silica nanoparticles loaded with IR780, both in vitro and in vivo. This implies a potential and encouraging approach for HCC treatment [61]. In 2023, A study demonstrated that modifying the hinge and transmembrane domains of a nanobody-based CAR, designed to target a remote GPC3 epitope, triggered potent T-cell signaling. This adaptation resulted in prompt and enduring elimination of HCC [62]. In 2024, Zhang Qi et al. reported the safety and anti-tumor activity of C-CAR031, an autologous CAR-T cell therapy targeting GPC3, in 24 patients receiving intravenous infusion. Among the 22 patients evaluated for efficacy, 90.9% experienced tumor shrinkage, with improvements in both intrahepatic and extrahepatic lesions, and a median reduction rate of 44.0%. The disease control rate across all cohorts was 90.9%, with an overall response rate (ORR) of 50.0%. In the DL4 cohort, the ORR was 57.1%. No dose-limiting toxicities or ICANS were observed, and low-grade CRS was noted in 22 patients [63].

### Disialoganglioside 2(GD2)

GD2 is a specific target for neuroblastoma immunotherapy. Several studies are currently underway in clinical trials. In 2020, Michelle Monje’s team from Stanford University initiated a phase 1–2 clinical trial which aimed to evaluate the feasibility and safety of GD2-CART01, a third-generation GD2-CAR-T cell therapy expressing the inducible caspase 9 suicide gene, in patients experiencing relapsed or refractory high-risk neuroblastoma [64]. In 2022, first-in-human phase I clinical trial involving GD2-CAR-T cells for the treatment of H3K27M-mutated diffuse intrinsic pontine glioma (DIPG) or spinal cord diffuse midline glioma (DMG) showed notable efficacy, among the four patients enrolled, three exhibited improvements both clinically and radiographically. This positive outcome was attributed to elevated levels of pro-inflammatory cytokines in both the plasma and cerebrospinal fluid (Fig. 4C and D) [64]. In 2023, Francesca Del Bufalo et al. released the clinical trial data for GD2-CART01 in the treatment of relapsed or refractory high-risk neuroblastoma. Six weeks after GD2-CART01 infusion, 9 out of 27 patients (33%) achieved a complete response (8 patients) or sustained a previously achieved complete response (1 patient) [64]. Research on combination therapy is also making progress. In 2023, a study indicated that administering a high dosage of GD2 CAR-T induced tumor apoptosis via the p53/caspase-3/PARP signaling pathway. This suggests that combining GD2 CAR-T with Nivolumab could present an enhanced therapeutic approach for glioblastoma treatment [65]. A phase 1–2 clinical trial conducted by Quintarelli, C, et al. investigated the efficacy of GD2-CART01 treatment in 27 pediatric neuroblastoma patients who had undergone multiple prior therapies. Within 30 months post-treatment, the overall response rate was 63% (17/27), which thus far constitutes the most promising data generated using CAR-T in the solid tumor setting., suggesting the safety and feasibility of GD2-CART01 therapy for high-risk neuroblastoma and the possibility of a prolonged anti-tumor effect [66].

### Human epidermal growth factor receptor 2(HER2)

**Fig. 5:**
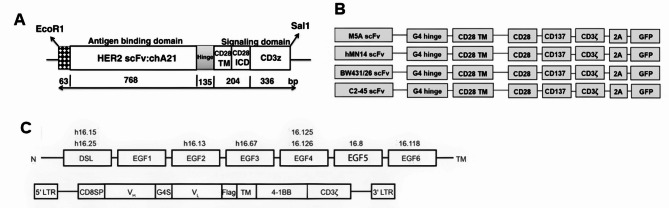
Structures of the CARs mentioned above. **(A)** Diagram of the HER2-specific CAR, consisting of a humanized chA21 single-chain variable fragment (scFv) linked to CD28 and CD3z signaling moieties [67]. Reproduced with permission. Copyright 2014, Sun et al.; licensee BioMed Central Ltd. **(B)** Structures of the 4 CEA-specific CARs with the corresponding scFvs [84]. Copyright 2023, by the authors, licensee CC BY 4.0. **(C)** Schematic of extracellular DLL3 domains with binding location of anti-DLL3 SC16 antibody clones (top), and schematic of CAR design for initial selection of single-chain variable fragments (bottom) [76]. Copyright 2023, Jaspers et al. Licensee CC BY 4.0. All these figures are not modified

HER2 is a member of the transmembrane epidermal growth factor receptor family, standing out as one of the extensively investigated tumor-associated antigens in the realm of cancer immunotherapy. It is frequently regarded as a specific target for ovarian and breast cancer (Fig. 5A) [67]. Research has demonstrated that HER2-CAR-T exhibits specific recognition of HER2-positive tumor cells, effectively inhibiting tumor growth both in vivo and in vitro. Furthermore, the therapeutic efficacy of HER2-CAR-T on tumors is significantly enhanced by anti-PDL1 treatment [68]. Another study highlighted that inhibiting PD1-mediated immuno-suppression can enhance the activation of CAR-T cells once they are activated by a targeting antigen, while third generation anti-HER2 CAR-T cells in combination with PD1 blockade showed considerable promise in treating malignant glioblastoma [69]. In 2020, Baylor College conducted clinical trials involving the infusion of autologous HER2 CAR-T cells for pediatric patients with refractory metastatic alveolar rhabdomyosarcoma. Following initial remission and subsequent relapse, patients underwent additional CAR-T cell reinfusion in combination with the PD-1 antibody pembrolizumab. Remarkably, they achieved complete recovery and remained relapse-free for the following 20 months, suggesting the effectiveness of CAR-T therapy for HER2-positive solid tumors [70]. In a phase I clinical study published in 2024, researchers investigated the safety and efficacy of HER2 CAR-T cell therapy in patients with advanced sarcoma. The trial included 14 eligible patients with HER2-overexpressing sarcomas. Among the treated patients, 50% experienced clinical benefit, with one osteosarcoma patient achieving complete remission (CR) after multiple CAR-T cell infusions, with the remission lasting over six years. Of the 12 patients, 9 experienced grade 1–2 cytokine release syndrome [71].

### Carcinoembryonic antigen (CEA)

CEA is associated with poor cancer prognosis and is targeted for the treatment of breast, lung, colorectal, gastric and pancreatic cancers [72]. In 2019, Chi et al. engineered CAR-T cells specific to CEA and employed them in conjunction with recombinant human IL-12 (rhIL-12) for the treatment of various solid tumors. Their findings confirmed that the concurrent use of cytokines such as rhIL-12 enhances the anti-tumor activity of CAR-T cells [72]. In 2023, Zhang et al. conducted a comparison of four humanized or fully human anti-CEA antibodies (C2-45, BW431/26, hMN-14, and M5A) utilizing a 3rd-generation CAR structure. Among these, M5A exhibited the highest levels of cell proliferation and cytokine secretion (Fig. 5B) [73]. Sato et al. assessed the correlation between the expression level of CEA and the antitumor efficacy of anti-CEA-CAR-T through a functional assay involving various pancreatic ductal adenocarcinoma (PDAC) cell lines and proved the correlation between tumor heterogeneity and the intensity of CEA immunostaining. Therefore, CEA expression levels can serve as clinically relevant biomarkers for the selection of PDAC patients eligible for anti-CAR-T therapy [74].

### Delta-like ligand 3(DLL3)

DLL3 has been identified as a distinctive cell surface marker exclusive to neuroendocrine cancers, observed in small cell lung cancer (SCLC) [74]. Zhang et al. proved that infusing DLL3 CAR-T cells exhibited strong anti-tumor efficacy, resulting in complete responses, in both subcutaneous and systemic in vivo models of SCLC [75]. In vivo experiments on mice also demonstrated that the production of IL-18 significantly enhanced the activation of CAR-T cells, antigen-presenting cells and endogenous tumor-infiltrating lymphocytes thus greatly enhancing the effectiveness of DLL3-targeting CAR T cell therapy (Fig. 5C) [76]. In 2023, Zhou et al. reported the clinical pharmacology profile of AMG119, the pioneering CAR-T cell therapy targeting DLL3, in individuals with relapsed/refractory (R/R) SCLC and obtained encouraging cellular kinetics data [77], bringing hope for the drug to market. Other CAR-T therapies in development include ALLO-213 and LB-2102.

### Guanylyl cyclase C(GUCY2C)

GUCY2C serves as a versatile biomarker and distinctive CAR-T therapeutic target for colorectal cancer [78]. Research shows the potential of human GUCY2C-targeted CAR-T cells eliminating colorectal cancer metastases [28]. In vitro experiments demonstrated that CAR-T cells targeting human GUCY2C exhibit a selective capacity to eliminate colorectal cancer cells expressing GUCY2C, concurrently inducing the production of inflammatory cytokines upon antigenic stimulation [79]. In 2024, Qi Changsong et al. reported a phase I clinical trial of CAR-T therapy for GUCY2C-positive metastatic colorectal cancer (mCRC). Among 19 evaluable patients, the disease control rate (DCR) was 73.7%, and the overall response rate (ORR) was 26.3%. In the DL3 cohort, the ORR was 40%, with a median progression-free survival (PFS) of 7 months and a median response duration of 10 months. A reduction in carcinoembryonic antigen levels correlated with tumor response [80]. Recently, Naifei Chen and colleagues published clinical study results of GCC19CART in metastatic colorectal cancer, showing an objective response rate (ORR) of 40% and a median overall survival (mOS) of 22.8 months. Responding patients had an approximately 75% chance of survival two years later [81].

### Epidermal growth factor receptor (EGFR)

**Fig. 6:**
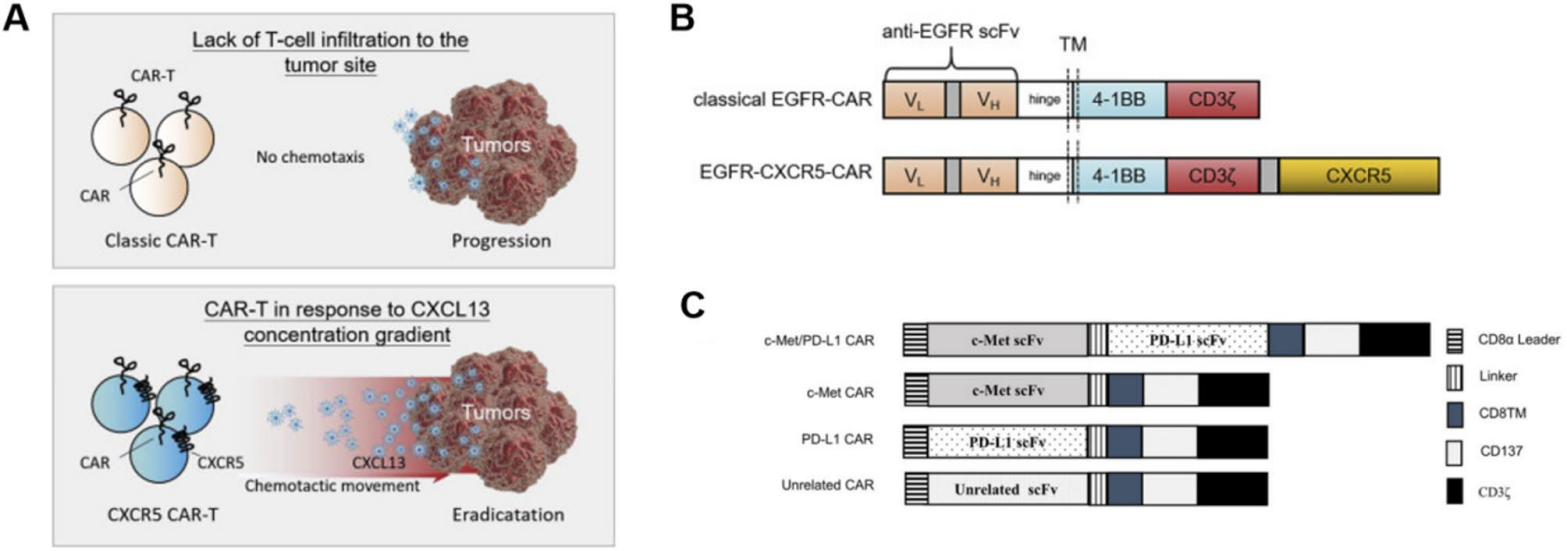
Mechanism of action of CAR-T cells and structure of CARs. **(A)** The motorized CAR-T cells could infiltrate into the tumor site along the gradient of CXCL13 to further clear the tumor cells when CAR-T cells are modified with the CXCR5 receptor [83]. Copyright 2021 The Authors. **(B)** Graphical representation of the CAR designed using the anti-EGFR scFv, CD8a hinge, and transmembrane domain, 4-1BB and CD3zeta endodomain [83]. Reproduced with permission. Copyright 2021 The Authors. (**C)** Schematic illustration of a lentiviral vector encoding CARs [83]. Copyright 2021 The Authors. All these figures are not modified

EGFR is closely associated with the development and advancement of solid tumors, establishing itself as a crucial therapeutic target, particularly in non-small-cell lung carcinoma (NSCLC), glioblastoma, colorectal, breast, gastroesophageal cancers and so on [82]. In 2021, Li et al. designed an EGFR CAR-T cell that incorporates a second receptor, CXCR5, with the aim of promoting the migration of CAR-T cells towards NSCLC tumors expressing CXCL13. This innovative approach suggests a novel method for enhancing T cell infiltration to the tumor site (Fig. 6A and B) [83]. In 2023, Li et al. demonstrated the effectiveness of TGF-β signaling-resistant EGFR-targeted CAR-T cells, achieved through SMAD7 overexpression specifically confined to the CAR-T cells themselves. EGFR-SMAD7-CAR-T cells exhibited an elevated proliferation rate and enhanced lysis capacity against carcinoma cells [84].

In addition, there are a number of dual-target and multi-target CAR-T therapies under research, such as c-Met/PD-L1 CAR-T cells for treating HCC(Fig. 6C) [85], co-express GPC3 with EGFR-targeted CAR-T treating HCC [86] Her2/B7H3 CAR-T cells treating CNS tumors [87] and so on. A multiple-target CAR-T cell therapy approach can enhance anti-tumor efficacy and mitigate the risk of relapse attributed to antigen loss [88]. Currently, several CAR-T drugs are undergoing clinical test both domestically and internationally.

### Claudin18.2 (CLDN18.2)

Claudin18.2 is highly expressed in multiple cancers such as lung mucinous adenocarcinoma, colorectal adenocarcinoma, and cervical adenocarcinoma [89], positioning it as a promising target for anti-tumor therapy. CT041 is a global first-in-class autologous CAR-T cell candidate product targeting Claudin18.2, designed for the treatment of Claudin18.2-positive solid tumors. It is primarily used for the treatment of gastric cancer [90] or pancreatic cancer [43]. In 2024, an analysis of two phase I/Ib clinical trials for the treatment of pancreatic cancer with CT041, conducted by the research team at Peking University Cancer Hospital, showed that among the 24 pancreatic cancer patients enrolled, 12 showed tumor target lesion shrinkage. The disease control rate (DCR) was 70.8%, with a median overall survival (mOS) of 10.0 months, a 1-year survival rate of 45.8%, a median duration of response (DOR) of 9.5 months, and a 1-year DOR rate of 50% [91]. In the same year, Qi et al. published the final results of a phase I clinical trial on CT041 for patients with Claudin18.2-positive advanced gastrointestinal tumors. Among the 98 patients, 70 exhibited varying degrees of tumor regression. In 51 patients with gastric cancer or gastroesophageal junction adenocarcinoma who had target lesions and were treated with CT041 monotherapy, the overall response rate (ORR) and disease control rate (DCR) were 54.9% (28/51) and 96.1% (49/51), respectively, with a median duration of response (mDOR) of 6.4 months. Among all gastric cancer/gastroesophageal junction adenocarcinoma patients receiving CT041 monotherapy (*n* = 59), the median progression-free survival (mPFS) was 5.8 months, and the median overall survival (mOS) was 9.0 months, with a 12-month survival rate of 37.3% [92].

### Novel antigen targets in CAR-T therapy for solid tumors

#### CS1 (SLAMF7)

CS1 is highly expressed in multiple myeloma (MM) and has demonstrated significant antitumor activity in preclinical models. It is currently being explored in clinical trials for MM. The advantages of targeting CS1 include effective reduction of tumor burden and prolonged survival in xenograft models [93]. However, there are several disadvantages associated with CS1-targeted CAR-T therapy. These include the potential for cytokine release syndrome (CRS), viral infections, and fratricide of normal lymphocytes, which can reduce the overall efficacy of the treatment. Additionally, the expression of CS1 on normal immune cells, such as CD8 + T cells and NK cells, poses a risk of “on-target, off-tumor” toxicity, potentially leading to prolonged immunodeficiency [94].

#### TEM8/ANTXR1

TEM8/ANTXR1 has been investigated as a therapeutic target in various cancers, including gastric adenocarcinoma, showing promising preclinical results. The advantages of targeting TEM8/ANTXR1 include its overexpression in tumor-associated endothelial cells and certain tumor cells, which can potentially enhance the specificity of treatments towards tumor tissues. However, there are also disadvantages. One major concern is the potential for off-target effects, as TEM8/ANTXR1 is also expressed in healthy tissues, which may lead to toxicity and other adverse effects, as demonstrated in some in vivo studies [95].

#### Glycoforms of antigens

Recent studies have delved into the glycoforms of antigens, with a particular focus on the cancer-associated Tn glycoform of MUC1. This glycoform is aberrantly expressed in a variety of cancers but is virtually absent on the surface of normal tissues. The research has demonstrated that CAR T cells engineered to target this specific glycoform exhibit robust anti-tumor activity in preclinical models of leukemia and pancreatic cancer, with minimal off-target effects. This highlights the potential of targeting unique glycosylation patterns as a novel and effective strategy in CAR-T therapy, offering a promising direction for the development of more specific and safer cancer treatments [96]. Another study shows that the cancer-associated Tn, T, and sialyl-Tn glycoforms of antigens such as MUC1 and CEACAM5. These aberrantly glycosylated forms are uniquely expressed on tumor cells but are virtually absent on the surface of normal tissues Table 2. CAR T cells engineered to target these specific glycoforms exhibit robust anti-tumor activity in preclinical models of various cancers, including leukemia, pancreatic cancer, and breast cancer, with minimal off-target effects Table 3. This focus on unique glycosylation patterns represents a novel and promising direction in CAR-T therapy, potentially offering a safer and more effective approach to treating solid tumors [97].

**Table 2:** Comprehensive clinical evaluation and pros and cons of antigen targets in CAR-T therapy for solid tumors

Antigen Target	Clinical Evaluation	Advantages	Disadvantages
GPC3	Highly expressed in HCC with immune evasion issues	Good antitumor activity and high disease control rate	Ineffective in some patients, immune escape
GD2	Specific target for neuroblastoma and other neuroendocrine tumors	Good tolerability, long-term remission in some patients	CRS and other side effects
HER2	Highly expressed in ovarian cancer, breast cancer, and other tumors	Good antitumor activity	CRS and other side effects, potential toxicity to normal tissues
CEA	Associated with poor prognosis in various cancers	Good antitumor activity	“On-target, off-tumor” toxicity, ineffective in some patients
DLL3	Specific cell surface marker for small cell lung cancer (SCLC)	Promising antitumor effects in preclinical studies	Limited clinical trial data
GUCY2C	Serves as a biomarker and therapeutic target for colorectal cancer	CAR-T therapy shows disease control rate and objective response rate in clinical trials	Limited clinical data, need for further studies to confirm long-term effects and safety
EGFR	A crucial therapeutic target in various solid tumors	Enhanced T cell infiltration by designing EGFR CAR-T cells with CXCR5	Need for further research to improve CAR-T cell infiltration and persistence in solid tumors
CLDN18.2	A promising target for anti-tumor therapy in multiple cancers	CT041, the first-in-class CAR-T product targeting CLDN18.2, shows disease control rate and objective response rate in clinical trials	Limited clinical data, need for further studies to confirm long-term effects and safety

Note: CRS = Cytokine Release Syndrome

**Table 3:** Consolidated table of clinical trials in CAR-T therapy for solid tumors

Target Antigen	Trial Identifier	Phase	Indication	Cell Design	Response Rate	CRS/ICANS Incidence
MSLN	CT041	I/II	Pancreatic Cancer	Second-generation CAR-T	ORR: 54.9% (28/51) DCR: 96.1% (49/51)	Not reported
GPC3	C-CAR031	I	Hepatocellular Carcinoma	GPC3-specific CAR-T	ORR: 50.0% (11/22) DCR: 90.9% (20/22)	Low-grade CRS in 22 patients
GD2	GD2-CART01	I/II	Neuroblastoma	Third-generation CAR-T	ORR: 63% (17/27) CR: 33% (9/27)	Not reported
HER2	N/A	I	Advanced Sarcoma	HER2-specific CAR-T	ORR: 50% (7/14) CR: 1 (1/14)	Grade 1–2 CRS in 9 patients
CEA	N/A	I	Colorectal Cancer	CEA-specific CAR-T	ORR: 30% (3/10) DCR: 70% (7/10)	Not reported
DLL3	AMG 119	I	Small Cell Lung Cancer	DLL3-specific CAR-T	ORR: 40% (4/10) DCR: 80% (8/10)	Not reported
GUCY2C	IM96	I	Metastatic Colorectal Cancer	GUCY2C-specific CAR-T	ORR: 26.3% (5/19) DCR: 73.7% (14/19)	Not reported
EGFR	N/A	I	Non-Small Cell Lung Cancer	EGFR-specific CAR-T	ORR: 30% (3/10) DCR: 70% (7/10)	Not reported
CLDN18.2	CT041	I/II	Gastric Cancer	Second-generation CAR-T	ORR: 54.9% (28/51) DCR: 96.1% (49/51)	Not reported

Note: ORR = Overall Response Rate, DCR = Disease Control Rate, CRS = Cytokine Release Syndrome, ICANS = Immune Effector Cell-Associated Neurotoxicity Syndrome

### Difficulties in CAR-T treatment of solid tumors

**Fig. 7:**
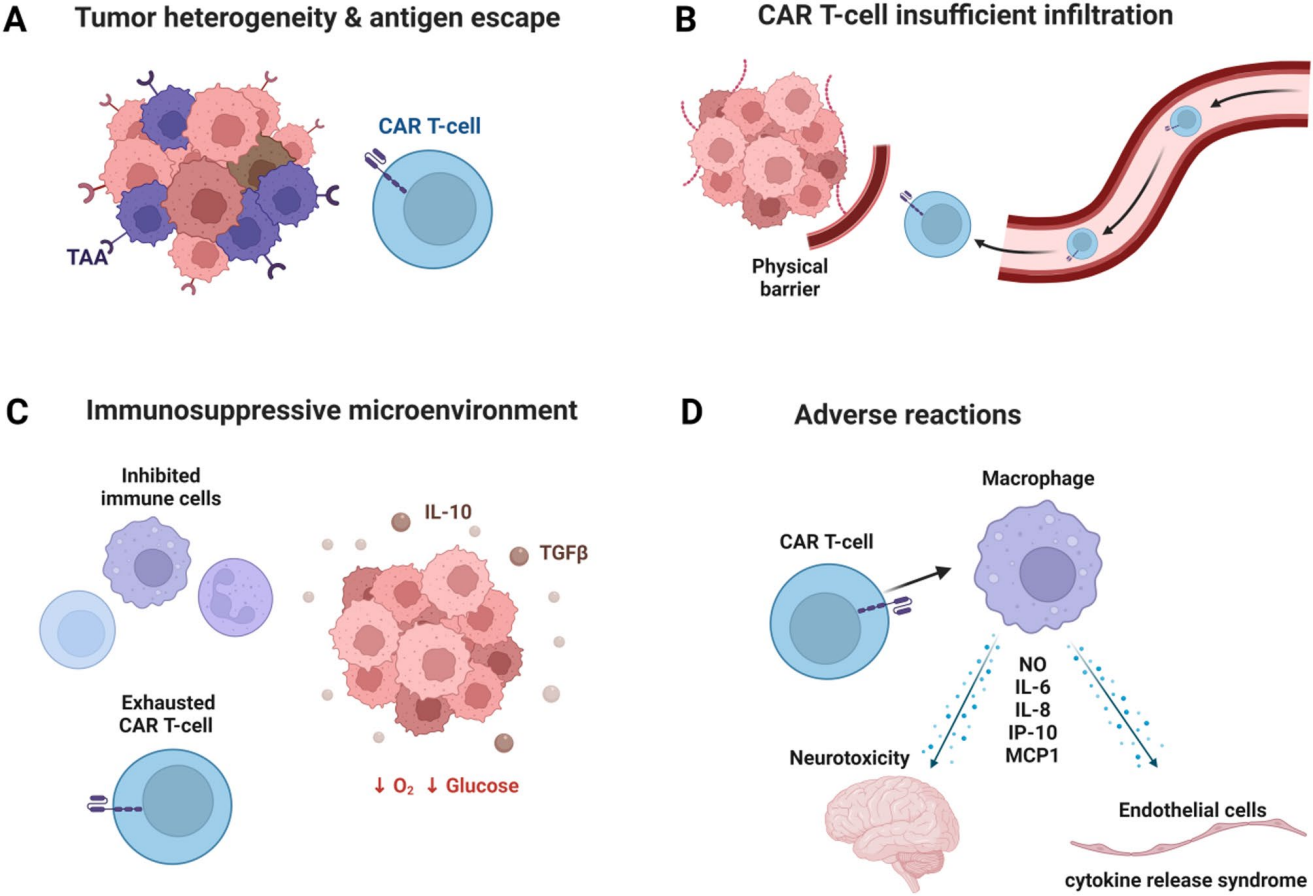
Challenges for CAR T-cell Immunotherapy in Solid Tumors. **(A)** The expression of tumor antigens shows significant heterogeneity between different patients or different cell populations. **(B)** Physical barriers obstruct CAR-T cell infiltration. **(C)** The distinctive immunosuppressive components of the tumor microenvironment diminish the efficacy of CAR-T cells. **(D)** Cytokine release syndrome and Immune Effector Cell-Associated Neurotoxicity Syndrome are the main adverse reactions of CAR-T therapy

Unlike treatments for hematological malignancies, CAR-T therapy still faces many new problems in the field of solid tumor treatment, including the heterogeneity of tumor antigens, insufficient CAR-T cell infiltration, poor proliferation and persistence, toxicity control, and immunosuppressive microenvironment (Fig. 7).

### Target antigen selection

Despite the rapid progress in detection technology, the identification of effective target antigens remains a gradual process thus far. The heterogeneity of tumor antigens is a key reason. The expression of tumor antigens is significantly different in different patients with the same type of tumor and different cell populations of the same tumor, which makes the screening of antigens difficult. Besides, one of the major challenges in ensuring target safety is to avoid the “on-target off-tumor” effect: non-specific expression of the target antigen on healthy cells can stimulate CAR-T cells, resulting in damage to normal tissues and posing a potential life-threatening risk to patients. The primary mechanism through which CAR-T cells induce damage to normal cells involves the release of perforin and granzymes. This process is coupled with the upregulation of T cell surface molecules, leading to target cell apoptosis or the secretion of cytokines. Therefore, in the pursuit of creating CAR-T cells that are both safe and efficacious for patients with solid tumors, the identification of new antigens is crucial. These antigens should be selectively expressed exclusively on malignant cells and absent in non-malignant cells. Measures currently available to address this problem include fine-tuning CAR domains, constructing Logic-gated CAR-T cells, control via suicide switches or by regulating cytotoxicity and CAR expression and local injection [98].

### Physical barriers

CAR-T may encounter barriers while entering some solid tumors, such as the blood-brain barrier (BBB) in treating CNS tumors. Traditional intravenous administration makes it difficult for CAR-T cells to pass through BBB. Hence, laser thermotherapy, electroporation, transcranial ultrasound and many other techniques have been explored to modify the BBB, which has been proposed as a potential avenue for the delivery of CAR T-cell therapy. Additionally, alternative methods such as direct delivery to the brain or the intraventricular system are being investigated to provide a targeted approach to therapy [99, 100]. It has been proved that intraventricular therapy delivery has the advantage of bypassing many obstacles within the brain parenchyma [101]. However, researchers found that BBB disruption can trigger CNS-related side-effects including brain swelling [102]. In addition to physiological barriers, the solid tumor itself also has a physical barrier. Activated cancer-associated fibroblasts contribute to the formation of thicker and mechanically stressed collagen fibers, providing structural support for tumor growth. Simultaneously, in solid tumors, the basement membranes often experience breaches facilitated by both proteolytic degradation and force-induced realignment of molecular components within the ECM [103], thus obstructing CAR-T cell infiltration.

### Antigen escape

Solid tumors exhibit significant antigen heterogeneity, and clinical studies have indicated a swift occurrence of antigen escape from therapy. This rapid escape curtails the persistence and effectiveness of CAR-T cells [87]. It can happen either through mutation of cancer cells or by proliferating from a cell without the antigen that existed from the time of treatment [104]. Therefore, CAR-T cells that target multiple antigens is an available method to refine on this challenge. CAR-T cells targeting two single antigens, in a double-antigen-targeted approach, effectively overcome the challenge of antigen escape and enhance the specificity for target antigens [105]. Common modes of dual-target CAR-T include combination of two CAR-T cells, bicistronic CAR-T cells and tandem bispecific CAR-T cells [106].

### Immunosuppressive microenvironment

In solid tumors, the distinctive immunosuppressive components of the tumor microenvironment (TME) play a role in the diminished efficacy of CAR-T cells. Previous studies have shown that merely 1–2% of CAR-T cells can finally infiltrate the core of the tumor, leading to a substantial reduction in killing efficiency [107].The heterogenous constituents of the TME include a diverse array of cells such as natural killer cells, tumor-associated macrophages [108], myeloid-derived suppressor cells, myeloid progenitor cells, effector and regulatory T cells, and dendritic cells, matrix proteins including an extracellular matrix comprised of proteoglycans, fibrous proteins, stromal cells, glycoproteins, and polysaccharides, and secreted factors like chemokines, cytokines, and other proteins [109], which can block CAR-T cells by means of forming immunosuppressive cell barrier, affecting the intricate interplay of signaling or altering immune cell direction. Moreover, the insufficient levels of nutrients and oxygen, coupled with the accumulation of metabolic waste, contribute significantly to the highly immunosuppressive nature of the tumor microenvironment [110]. Additionally, the presence of abnormal vascular beds and elevated interstitial fibrosis in solid tumors hampers the effective delivery of CAR-T cells or drugs to the deeper regions of the tumor [111]. Co-Treatment with immune checkpoint inhibitors might be an outstanding means to overcome this challenge [112].

### Adverse reactions

**Fig. 8:**
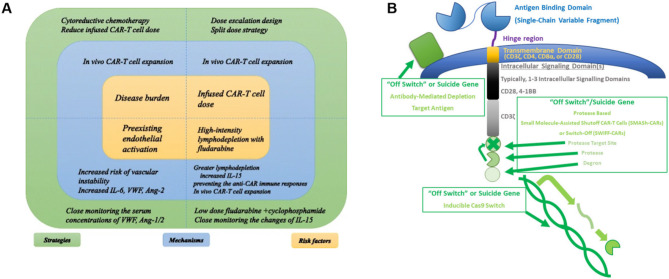
Adverse reactions and solutions. **(A)** Risk factors for CRS and neurotoxicity [113]. Copyright 2018 All the authors. Licensee http://creativecommons.org/licenses/by/4.0/. **(B)** A few types of “off switches” or suicide genes can be manipulated to alter the behavior of CAR-T cells by controlling whether CAR-T cell receptors are degraded or are able to be expressed [115]. Copyright 2022 Sterner and Sterner. Licensee CC BY 4.0. All these figures are not modified

CAR-T cell therapy may cause serious adverse reactions, mainly cytokine release syndrome (CRS) and neurotoxicity, which can occur during the treatment of either solid or non-solid tumors (Fig. 8A). CRS stems from the excessive production of inflammatory cytokines induced by supraphysiological levels of immune activation. This can manifest as a clinical constellation of severe symptoms, such as fatigue, nausea, fever, muscle pain, low blood pressure, general discomfort, reduced oxygen levels, blood clotting disorders, capillary leakage, or multiorgan dysfunction, and may pose a risk of lethality [113]. Drugs like tocilizumab alone or with steroids are applied to severe CRS [114]. Immune Effector Cell-Associated Neurotoxicity Syndrome (ICANS) is a neurotoxicity related to CAR-T cell therapy that has the potential to be life-threatening [115], which can trigger symptoms like delirium, aphasia, encephalopathy, seizures and tremor, and in rare cases, rapid-onset cerebral edema [116]. Possible pathogenesis includes BBB disruption and specific production of IL6, IL8, IP10, and MCP1 [117]. When constructing new generations of CAR, the mere incorporation of a co-stimulation domain may lead to the emergence of severe side effects. To mitigate these complications, pro-apoptotic suicide gene including iCaspase9, has recently been integrated in some researches so that cytotoxic injuries and systemic effects can be terminated in time (Fig. 8B) [118].

## Discussion

If CAR-T cells continue to face challenges in treating solid tumors, several alternative novel immunotherapeutic strategies could be considered. These include bispecific antibodies (BsAbs) that redirect T cells to tumor cells, antibody-drug conjugates (ADCs) that deliver cytotoxic drugs directly to cancer cells, oncolytic viruses that selectively infect and lyse tumor cells, immune checkpoint inhibitors that block inhibitory signals and enhance T cell activity, cancer vaccines that stimulate the immune system to recognize and attack cancer cells, natural killer (NK) cells that can be engineered to target tumors, macrophage-targeted therapies that reprogram tumor-associated macrophages, and combination therapies that integrate multiple strategies to address various aspects of tumor immunology. More detailed research on these and other immunotherapeutic approaches can be found in other immunology articles and will not be reiterated here. Each of these approaches has shown promise in preclinical and early clinical studies, and their continued development could lead to significant advancements in the treatment of solid tumors.

## Summary

CAR-T cell therapy in solid tumors is gradually maturing, and related technologies are flourishing. For the moment, the tumor microenvironment and the intrinsic factors of T cells are the main reasons for the poor efficacy of CAR-T cells. Researchers have used genetic engineering techniques to modify CAR-T cells to break through these barriers and increase the function of CAR-T cells. At present, CAR has been built to the fifth generation, and it is certain that there will be a newer generation of CAR in the future to address the shortcomings of CAR-T therapy in clinic from the structure of the cell itself, including designing updated scFv fragments, adding new fragments in the intracellular domain, knocking out or modulating immunosuppressive targets, etc. to achieve the goal of controlling adverse reactions such as cytotoxicity, enhancing cell proliferation ability, strengthening cell targeting ability and overcoming immunosuppressive environment. Furthermore, the potent combination with IL-2 receptors, co-stimulatory molecules, TCR signaling complexes, and chemokine receptors to enhance CAR-T receptor signaling and improve CAR-T cell migration into hostile tumor microenvironments is also a direction worth considering for next generation of CAR.

In addition, new therapeutic modalities such as nanocarriers [119], cancer vaccines [120], dual targets and cytokine combination therapy have also greatly enhanced the efficacy of CAR-T, and achieved remarkable results in clinical trials. It is foreseeable that newer CAR-T treatment models will be emerge in the future, and the existing technology will be combined with CAR-T to play a better role.

As a burgeoning cancer treatment, CAR-T cell therapy in solid tumors has a prosperous future. At present, CAR-T therapy has already achieved some success in the treatment of solid tumors. Although it still faces some challenges, such as high treatment cost and some side effects, it is believed that with the continuous innovation and improvement of technology, CAR-T will have broad potential for development in the field of solid tumor treatment because of its outstanding clinical efficacy and technology specificity and will also make greater contributions to the anti-tumor cause.

## Data Availability

No datasets were generated or analysed during the current study.
